# Same-Day Discharge After GreenLight Laser Photoselective Vaporization of the Prostate in Patients With Moderate to Severe Lower Urinary Tract Symptoms

**DOI:** 10.7759/cureus.112408

**Published:** 2026-07-10

**Authors:** Mohamed Abouelenein, Noman Ali Ghazanfar, Gausal Manoharan, Abdur Rasheed, Omer Onur Cakir, Sanjith Gnanappiragasam

**Affiliations:** 1 Urology, King's College Hospital NHS Foundation Trust, London, GBR; 2 Urology, Central Park Medical College and Teaching Hospital, Lahore, Lahore, PAK; 3 Urology, University College London Hospitals NHS Foundation Trust, London, GBR

**Keywords:** benign prostatic hyperplasia (bph), endourology, greenlight laser prostatectomy, greenlight laser pvp, same day discharge

## Abstract

Background: The most common cause of severe lower urinary tract symptoms (LUTS) in men over 50 is benign prostatic hyperplasia (BPH). A wide range of day-case surgical options are practiced, among which GreenLight Photoselective Vaporization of the Prostate (GL-PVP) is one. This has completely taken over conventional bipolar transurethral resection of the prostate (TURP). There is a need to study whether in patients with moderate and severe International Prostate Symptom Score (IPSS) scores, GL-PVP can give safe and effective outcomes as a same-day discharge (SDD).

Objective: To study the safety and effectiveness of GL-PVP as SDD in patients with moderate and severe LUTS.

Method: The study was conducted at King's College Hospital, United Kingdom (UK). Patients presenting with severe LUTS on IPSS and quality of life (QOL) scoring were included. All patients had their pre-operative IPSS + QOL scoring and flow rate measured by uroflowmetry in the clinic. They underwent GL-PVP and were discharged on the same day safely. Following this post-op, IPSS and flow rate on uroflowmetry were measured in the first follow-up.

Results: A total of 103 patients were included in the study, with prostate size ranging from 30 cc to 124 cc. The average laser time during GL-PVP was 21 mins, with an average energy used of 131,154 J. The mean improvement in IPSS + QOL score was from 25 ± 4 to 11.89 ± 1.61. The mean improvement in Q-max was from 9.81 ml/sec to 15.11 ml/sec. The mean improvement in post-void residual (PVR) volume was from 266 ml to 74.19 ml. None of the patients required secondary admissions or second procedures.

Conclusion: There was a marked improvement in IPSS + QOL scoring along with flow rate and PVR after SDD from GL-PVP prostate, proving efficacy and safety. This can be the alternative surgical option for patients with severe LUTS.

## Introduction

Benign prostate enlargement is a common cause of severe lower urinary tract symptoms (LUTS) in men over 50. It affects quality of life (QOL), necessitating surgical procedures in patients who fail to respond to medical therapy [1]. Among the wide range of armamentarium for bladder outlet obstruction (BOO) surgery, GreenLight Photoselective Vaporization of the Prostate (GL-PVP) has emerged in the last decade, evolving as an alternative to the most widely adopted conventional transurethral resection of the prostate (TURP) [1,2]. Initially thought of as a procedure for high-risk patients with bad cardiac profiles or bleeding disorders [3], GL-PVP is now a standard treatment option for all ranges of patients as a day case option in patients with severe LUTS, ensuring safety and efficacy [4].

There are several subjective and objective ways to determine the success of a GL-PVP procedure. IPSS scoring, along with QOL scoring, gives us immediate patient feedback on surgery, including improvement in his QOL, whereas measurement of post-void residual (PVR) by bladder ultrasound and measurement of flow rate on uroflowmetry are objective [5,6]. 

A number of studies over the past few years have contributed to the fact that GL-PVP is considered safe for outpatient surgery, with readmission and complication rates being low even for large cohorts in single centers and multicenters [4,7]. This provides an opportunity to focus on GL-PVP as a safe option for same-day discharge (SDD) and for those with severe symptoms. Assuming that this is shown to be effective in this population, GL-PVP can also be expected to decrease the need for conventional bipolar TURP, which involves hospitalization, bladder irrigation, and its associated potential complications [8,9]. It is also expected that a same-day GL-PVP pathway will be less costly for the healthcare system and greatly enhance patient experience. Therefore, the purpose of this study is to critically analyze and measure the safety of GL-PVP as a day-case option.

## Materials and methods

Study design and study setting

It was a retrospective study conducted after approval from the Institutional Ethical Review Committee of King’s College London NHS Hospital (Approval # 26/LO/8780). Patients were admitted with moderate to severe LUTS as a result of benign prostatic hyperplasia (BPH). In total, 103 patients participated in the study.

Inclusion and exclusion criteria

Patients were males aged 50 to 85 years who had a first-time BPH diagnosis with either moderate or severe symptoms on the International Prostate Symptom Score (IPSS) evaluation. Recovery data were only collected for patients who were undergoing GL-PVP and for whom recovery was planned through SDD.

Patients who had any previous surgical procedures that could lead to BOO or needed an adjuvant procedure simultaneously with BOO surgery; patients for whom clinical suspicion of prostate cancer was present or clinical signs of a neurological etiology for LUTS were present; those who declined to participate in the study; and those who were not considered stable enough to have the SDD due to underlying comorbidities were excluded from the study.

Baseline assessment

In addition to the routine baseline investigations, transabdominal or transrectal prostate ultrasound to assess prostate size was performed on all who were eligible. Each patient was then given a standard score sheet on the day of the outpatient appointment to document both preoperative IPSS [10] and QOL scores [11]. Uroflowmetry was also carried out in the clinic as a baseline measurement for maximum flow rate (Qmax) prior to surgery.

Surgical procedure and post-op care

All patients had GL-PVP and were all discharged home the same day. All patients were catheterized for a fixed period of 48 hours, deliberately determined to minimize procedure-related morbidity and also to reduce the interference in patients' QOL in the immediate postoperative period.

Patients were given an option after catheterization to either have a self-trial without a catheter (TWOC) that is supported by a patient information leaflet or to attend the nurse-led TWOC clinic at the end of 48 hours. Selection of either pathway was at the discretion of the individual patient.

Follow-up and outcome assessment

All patients were reassessed by the operating surgeon at the first postoperative follow-up visit and had repeated IPSS and QOL scores and uroflowmetry performed by the nurse. During this visit, patients were then educated on their post-op IPSS findings and flow rate.

Outcome assessment is assessed by comparing pre-operative and post-operative parameters, which are IPSS with QOL score, maximum flow rate (Qmax), and PVR volume on uroflowmetry. IPSS and QOL scoring represented the subjective patient-reported assessment of outcome, and objective measures of safety/efficacy of GL-PVP as an SDD procedure represented Qmax and PVR [10].

Statistical analysis

Continuous variables (Q-max, PVR, IPSS, and QOL) were compared pre- and post-operatively using a paired t-test. Statistical significance was defined as p < 0.05 with 95% confidence intervals (CI) calculated for all primary outcome improvements. All analyses were performed using IBM SPSS Statistics for Windows, Version 28 (Released 2021; IBM Corp., Armonk, New York, United States) or R software (R Foundation for Statistical Computing, Vienna, Austria).

## Results

A total of 103 patients were included in the study, with ages ranging from 50 to 85 years. The majority of patients (45.63%) were within the 60-70 year age group, followed by the 70-80 year age group (34.95%). Patient age distribution is summarized in Table 1. 

**Table 1:** Age demographics of included patients, along with respective percentages of the total sample size.

Age (years)	Number of Patients Included (% of total sample size)
50 to 60	13 (12.62%)
60 to 70	47 (45.63%)
70 to 80	36 (34.95%)
81-85	7 (6.79%)

The prostate size ranged from 30 cc to 124 cc on prostate ultrasound, ± 24.74 cc. A different amount of laser energy was required in each procedure, depending on the laser time and the size of the prostate. Their particular demographics were presented in Table 2.

**Table 2:** Classification of patients according to prostate size on preoperative ultrasound (cc), along with respective percentages of total sample size. "cc" stands for cubic centimeters

Size of Prostate on Ultrasound (cc)	Number of Patients Included (% of total sample size)
< 30 cc	17 (16.5%)
30 – 50 cc	29 (28.15%)
51 – 80 cc	40 (38.83%)
81 – 100 cc	8 (7.76%)
> 100 cc	9 (8.73%)

The amount of laser energy delivered during GL-PVP varied according to prostate size and operative time. Energy delivered ranged from less than 50,000 J to 350,000 J, with the largest subgroup of patients (28.15%) requiring between 100,001 and 150,000 J. The distribution of energy usage is shown in Table 3.

**Table 3:** Classification of patients according to the amount of energy used in Joules for the GL-PVP procedure, along with respective percentages of the total sample size. "J" stands for Joules

Total Energy Used in Joules During GL-PVP	Number of Patients Included
0 – 50,000 J	6 (5.82%)
50,001–100,000 J	23 (22.33%)
100,001–150,000 J	29 (28.15%)
150,001–200,000 J	13 (12.62%)
200,001–250,000 J	13 (12.62%)
250,001–300,000 J	11 (10.67%)
300,001–350,000 J	7 (6.79%)

Functional and symptomatic outcomes

The selected procedure used in all was GL-PVP, with a mean laser time of 21 mins ± 12 mins and a mean energy given during the procedure of 131,154 joules. None of the patients required secondary admissions or second procedures. All patients had successful removal of the catheter after 48 hours. 

The mean pre-operative Q-max was 9.81 ml/sec with a mean post-operative Q-max of 15.11 ml/sec, with an improvement of 5.3 ml/sec. The mean pre-operative PVR was 266 ml, with a mean post-operative PVR of 74.19 ml, with an improvement of 191.81 ml. The mean preoperative IPSS + QOL score was 25 + 4, with a mean postoperative score of 11.89 + 1.61, with an improvement of 13.11 in the IPSS score and an improvement of 2.39 in the QOL score. All improvements reached statistical significance on paired t-test analysis (Table 4). 

**Table 4:** Clinical parameters comparison including Q-max, IPSS score, QOL score, and PVR. Q-max: maximum urinary flow rate; IPSS: International Prostate Symptom Score; QOL: quality of life; PVR: post-void residual

Metric	Preoperative Mean	Postoperative Mean	Absolute Improvement/Relative Change in %	Estimated 95 % CI	p- value	Statistical Test Used and T-value
Q-max (ml/sec)	9.81 ml/sec	15.11 ml/sec	+ 5.3 ml/sec (Relative Change in % = +54.0%)	4.25 to 6.35	< 0.001	Paired t-test, t-value 9.84
IPSS	25	11.89	- 13.11 points (Relative Change in % = -52.4%)	11.95 to 14.27	< 0.001	Paired t-test, t-value 22.09
QOL	4	1.61	-2.39 points (Relative Change in % = -59.8%)	2.05 to 2.73	< 0.001	Paired t-test, t-value 13.78
PVR (ml)	266 ml	74.19 ml	191.81 ml (Relative Change in % -72.1%)	168.40 to 215.22	< 0.001	Paired t-test, t-value 16.05

## Discussion

Management of LUTS in men over 50 years old is essential due to its prevalence and impact on QOL. BPH is the reason behind LUTS in men unless proven otherwise. After failure of medical therapy and lifestyle measures, BOO surgery is the next step in management. Options for BPH surgery include conventional bipolar TURP, Holmium Laser Enucleation of the Prostate (HOLEP), GL-PVP, and other minimally invasive surgical therapies (MIST) [12,13]. 

GreenLight laser PVP initially started for high-risk profile patients [14]; GL-PVP is ever-increasing and becoming favorable among endourologists around the world, replacing bipolar TURP. In this study, significant improvement in parameters was seen with GL-PVP, along with day-case discharges, eliminating the need for bipolar TURP and hospital admissions. 

Worldwide, different studies have been conducted for GL-PVP and its success as a potential replacement for bipolar TURP. A very good study was conducted by Yim et al. from the Department of Urology, Austin Health, Heidelberg, Victoria, Australia, where they compared HOLEP and GL-PVP for the long term, with both showing long-term success and safety profiles in patients [14]. 

There was a rather large study conducted in the United Kingdom (UK) by the Department of Urology, Victoria Hospital, NHS Fife, where they included more than 500 patients and proved GL-PVP as a day case treatment [15], similar to our study, where we are advocating the same-day care management of BPH disease by GL-PVP.

Castellani et al. from Italy gave a meta-analysis of photovaporization versus TURP, showing that GL-PVP has better perioperative (catheterization time, length of hospitalization, and blood transfusions) and early functional outcomes, supporting what we are advocating in our research, not only proving GL-PVP as a safer option but also showing superiority over conventional bipolar TURP [16]. 

Our study is different from a few in the sense that it not only addresses the GL-PVP as a day case but also includes patients with moderate to severe LUTS based on IPSS scoring, along with QOL scoring. The scoring is comprehensively described by Pandolfi et al. in their research from Italy [17] and also in many other urology textbooks as a well-recognized and most widely used scoring system for LUTS associated with prostate enlargement. Our study also includes very large prostate sizes approaching 125 cc. Including QOL scoring in our research proves GL-PVP a subjectively beneficial procedure, representing patient feedback experience through numbers. The concept of self-TWOC, 48 hours after the procedure, by providing a patient information leaflet after GL-PVP, was also included in our study, making it different from recent similar studies. We included both subjective and objective assessments in follow-up to give us a comprehensive analysis of the outcome after GL-PVP.

The limitation of our study, however, is the sample size of 103 patients. We are planning to conduct even larger data studies in the future. In the future, we also need to compare the other techniques, including HOLEP, MIST, and conventional TURP, through the same parameters and subjective patient analysis. We will address the above issues in our future research at King's College London. 

## Conclusions

There was a marked improvement in IPSS + QOL scoring, along with flow rate and PVR after SDD from GL-PVP, proving efficacy and safety. This can be a surgical option for patients with moderate to severe LUTS. This can also prove to be an alternative to conventional bipolar TURP surgery, reducing the workload on healthcare services by doing SDD. 
